# The exam performance of medical students with dyslexia: a review of the literature

**DOI:** 10.15694/mep.2017.000116

**Published:** 2017-07-03

**Authors:** Sebastian C. K. Shaw, Muzaffar Malik, John L. Anderson

**Affiliations:** 1 Brighton and Sussex Medical School

**Keywords:** Dyslexia, Specific Learning Difficulties, Neurodiversity, Literature Review, Medical Students

## Abstract

This article was migrated. The article was marked as recommended.

Introduction:

Dyslexia is a common condition. Estimates suggest it effects approximately 10% of the worldwide population, and 1.7% of UK medical students. This review aimed to explore the existing literature concerning the exam performance of medical students with dyslexia.

Methods:

A Review of Medline, ERIC, PsychInfo, The Cochrane Library, and Google Scholar was conducted in accordance with the PRISMA checklist. Papers were accepted if they concerned the exam performance of medical students with dyslexia.

Results:

Three papers were selected for review. These were all cross-sectional studies comparing exam results in students with dyslexia and without dyslexia - and the impacts of extra time in exams. A risk of bias assessment determined that all three were appropriate to include in this review. A meta-analysis was planned but could not be performed because the number of studies was low and heterogeneity between the studies too high.

There was consensus that Multiple Choice Question exams were fair for students with dyslexia, when extra time was allowed. Essay type exams were found to be particularly challenging for students with dyslexia. Students with dyslexia were also found to be at a disadvantage in their first year.

Discussion:

Overall, the evidence suggests that medical students with dyslexia are slower to adapt to medical school and under-perform early in the course. However, with appropriate supports, they appear to perform on a par with their non-dyslexic peers as they progress through their course.

Our review highlights the need for more research in the medical student population.

## Introduction

Dyslexia can be defined as “a learning difficulty that primarily affects the skills involved in accurate and fluent word reading and spelling.. It is best thought of as a continuum, not a distinct category, and there are no clear cut-off points” ( Rose J, 2009). It is classified as a Specific Learning Difficulty (SpLD) ( Wray et al., 2012). In accordance with the Equality Act (2010), employers and educators therefore have a duty to make ‘reasonable adjustments’ to ensure that people with dyslexia are not unfairly disadvantaged or discriminated against ( Great Britain, 2010).

Dyslexia is a common condition within the United Kingdom, and estimates indicate that up to 10% of the worldwide population have dyslexia ( Siegel, 2006). As medical schools strive to admit students from a wider, more representative, spectrum of society, it is not surprising that students with dyslexia (SWD) would be joining their ranks.

In 2009, the British Medical Association (BMA) estimated that 1.7% of medical students reported having an SpLD and that this number may be on the increase ( British Medical Association, 2009). A recent report suggested that, in Brighton and Sussex Medical School, 10% of their medical student population have SpLDs ( Mason et al., 2013). A commitment to Widening Access may have increased the prevalence over the past five years.

Our aims were twofold. Firstly we aimed to explore the effect, if any, of dyslexia on the exam performance of UK medical students. Secondly, we aimed to identify whether supportive measures made a difference on exam performance.

## Methods

In order to identify relevant research, Medline, ERIC, PsychINFO, The Cochrane Library and Google Scholar were searched. No time, language or location restrictions were used. Reference searching was also conducted in an attempt to identify further relevant studies. In an effort to ensure that relevant studies were not missed, search terms were kept broad. Combinations of “medical students”, “healthcare students”, “medical school” and “dyslexia” were used - along with wildcarding and MeSH Terms. Searches were performed up to February 2017. Using the PICOST criteria, our inclusion criteria were as follows ( Ho et al., 2013 May):

• Population - medical students with dyslexia;
• Intervention - N/A;
• Comparison - none, or medical students without dyslexia;
• Outcome Measures - exam performance;
• Study design - any;
• Timing - no restrictions.

**Figure 1.**
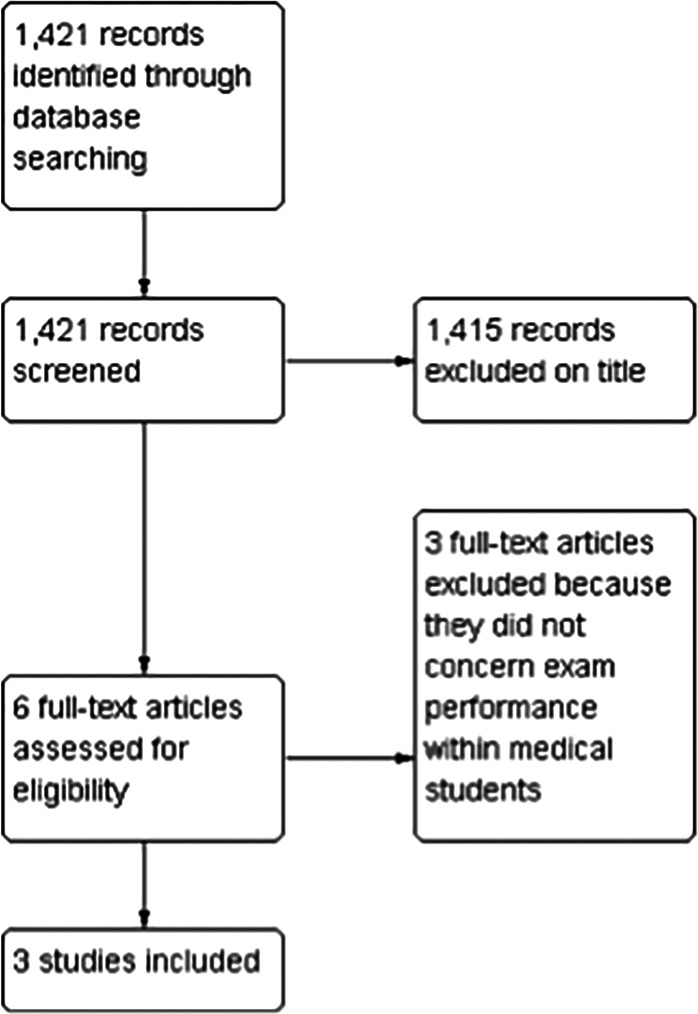
Study Flow Diagram

Our exclusion criterion was studies not matching the above inclusion criteria. The PRISMA checklist was used to facilitate the production of this review (Moher et al., 2009). Our PRISMA flow diagram can be seen in Figure 1 (Moher et al., 2009).

The authors developed a standard data extraction form. Data extraction was carried out by two of the authors independently (SS & JA) and cross-checked for concordance. Papers were assessed for risk of bias independently by two authors (SS & MM), using the Crowe Critical Appraisal Tool (CCAT) ( Crowe and Sheppard, 2011). RevMan was used to carry out a limited meta-analysis. A narrative analysis was then performed to compare the data from the three studies.

## Results

### Data Extraction

Three published (UK) studies were identified ( Table 1). Their References were searched, but no additional relevant studies were identified. Two (US) papers concerning medical students with dyslexia were rejected following quality assessment. A third study was rejected, as it concerned the lived experiences of junior doctors with dyslexia.

The three selected papers all adopted cross-sectional approaches and were conducted in the UK. They focussed on exam performance in medical students and the impact of remedial measures to support SWD within these exams. They analysed different exams, with different supportive measures, in students from different mixtures of academic cohorts. They were all single-site studies. Table 1 summarises the data.

**Table 1.** Data Extraction.

	Study		
	Ricketts et al.(2009)	Gibson & Leinster (2011)	McKendree & Snowling (2011)
Medical School	Peninsula	East Anglia	Hull York
Condition	Dyslexia & other SpLDs	Dyslexia	Dyslexia
Number with Dyslexia/SpLD*	50 & 40	91	36
Other Students	706 & 796	777	544
Percentage with Dyslexia*	7% and 5%	12%	7%
Years of study	1 - 5	1 - 4	1 - 2
Exams Included	MCQ - - - -	- EMQ - SAQO SCE	MCQ/EMQ combined As above MEQ - OSCE
Allowances made	25% extra time	25% extra time for written exams	25% extra time for written exams
Type of study	Cross-sectional	Cross-sectional	Cross-sectional
Method	Review of exam scores	Review of exam scores	Review of exam scores
Aims	To investigate the impact of dyslexia on medical school performance, and whether the 25% extra time adequately compensated for disability-specific difficulties	To examine: The impact of SpLDs on different assessment types (written and skills-based); The impact of dyslexia on performance;The difference made by adjustments (25% extra time).	To study the impact of dyslexia on performance in different exam formats.
Main Results	The extra time in MCQs allowed students with SpLDs to perform as well as others.	Without adjustments, SWD did less well (especially in SAQ) than others. Adjustments did not influence dyslexic students’ results in 1 ^st^ year EMQs. SWD did less well in OSCEs in 1 ^st^ Year but did as well later. Giving extra time in written exams had a positive effect.	All of the exam types studied could be completed (with adjustments) just as well by SWD as others. A variety of assessment types should be included in medical student assessments. No differences in scores on performance-based OSCEs.
Additional Issues Highlighted	In addition, MCQs are acceptable to most students with SpLDs and seemed fair.	It is important to conduct research into students’ attitudes to assessment types. Further research is needed into the suitability of support packages.	Although high performing SWD - “compensated dyslexics” - have developed some coping skills, they may be more anxious than others and require extra emotional support.

### Risk of Bias Assessment

In selecting the tool for a risk of bias assessment for cross-sectional studies, we browsed NICE guidelines, Cochrane guidelines, Evidence Based Medicine, the Critical Appraisal Skills Programme tools, and other tools for risk of bias assessment. No tools were available from these sources. We then looked at other sources and were only able to identify one. This was the CCAT from Dr Michael Crowe at James Cooke University, Australia ( Crowe and Sheppard, 2011). Using this tool we assessed the risk of bias within the papers and found the following. Two assessors independently agreed that all three studies were deemed sufficiently rigorous to review.

*Ricketts et al’s* abstract was vague about the number of separate participants within their study - “over 900” ( Ricketts et al., 2010). This was not clarified within their full text, instead referring to the figures highlighted above in Table 1. In order to gain a full understanding of the paper, the full text had to be read. The full text itself was clear, with a good search and presentation of the background literature and a good discussion of where their study fits into the existing literature. Ethical approval was “covered by a general ethical approval for medical education research granted by our medical school’s Research Ethics Committee” ( Ricketts et al., 2010). The discussion was logical and insightful. Clear and accurate conclusions were drawn. Study limitations were clearly discussed.

*Gibson & Leinster’s* abstract made no reference to the methodology or exact methods of the study. The full text, however, was sound. Background literature was appropriate; clear aims were stated, methods were well explained and appropriate statistical tests were applied. The discussion was easy to read and was appropriate. Study limitations were clearly discussed. Ethical approval was appropriate.

*McKendree & Snowling’s* abstract was clear and well subheaded. All sections of the abstract were well explained and logical. They contained appropriate background literature and stated clear aims within the introduction. Methods were explained in an overly complex way that made them difficult to understand. However, after detailed scrutiny, these were logical, and appropriate statistical tests were employed. Some data referred to within their report were, however, not presented within their tables. The discussion was appropriate, but lacked much reference to the wider literature. There was no clear discussion of study limitations. Ethical approval was appropriate.

### Meta-analysis

We planned to perform a meta-analysis of the comparable papers, for Multiple Choice Question (MCQ) and Extended Matching Question (EMQ) exams, from Ricketts et al. and Gibson & Leinster’s studies. However, the level of heterogeneity was found to be very high (I ^2^=92%) indicating that any meta-analysis could be misleading. We could not see any obvious causes for this difference, nor could we carry out any subgroup analyses, because there were only two studies. Likewise, we were unable to perform a meta-analysis for Objective Structured Clinical Examinations (OSCEs), as Gibson & Leinster did not provide their data on this assessment method. We therefore report a narrative summary below.

### Narrative Summary

Ricketts et al. (2009, N = “over 900”) found that SWD or other SpLDs performed equivalently to other students within MCQ exams when special adjustments were in place (Ricketts et al., 2009). They only included data from students with “a complete assessment record across the academic year..” and did not restrict the data to dyslexia-only, but presented them as “dyslexia / other SpLD” ( Ricketts et al., 2010). It is therefore unknown if poorer performing SWD dropped out or did not complete the academic years, or if the results were indeed representative of dyslexia as a stand-alone condition - as opposed to SpLDs in general. This may have altered the final results. It is also unclear why they chose to remove participants who had not declared their ethnicity from their analysis. Their aim was to explore the effect of dyslexia/SpLDs, not ethnicity.

Gibson & Leinster (2011, N = 777) compared the performance of medical SWD and medical students without dyslexia in EMQs, Short Answer Questions (SAQs) and OSCEs ( Gibson and Leinster, 2011). Their findings supported those of Ricketts et al. - finding similar performances between SWD and students without dyslexia in MCQ exams ( Gibson and Leinster, 2011). They, however, found that SWD tended to under-perform within both EMQs and SAQs ( Gibson and Leinster, 2011). This underperformance was only present within the first-year students, suggesting that SWD may just take longer to adapt to the academic requirements of the degree ( Gibson and Leinster, 2011). It is interesting to note that the poorer performance of SWD, compared to the rest of their cohorts, was also apparent in OSCEs (ANOVA, P < 0.05) ( Gibson and Leinster 2011). They found that SWD performed more poorly in data interpretation stations, and stations requiring the interpretation of clinical imaging (chi squared, P < 0.01) ( Gibson and Leinster 2011).

McKendree & Snowling (2011, N = 544) studied the impact of dyslexia on student performance within MCQs, EMQs, Modified Essay Question (MEQs) and OSCEs ( McKendree and Snowling, 2011). They found that SWD with special support measures currently in place - extra time in written assessments - did not under-perform in any of these assessments and, therefore, did not require further adjustments ( McKendree and Snowling, 2011). They also acknowledged the current lack of research into the effectiveness of non-MCQ assessment methods for SWD: “we hope that this study will be the first of many to look at a wider range of examination types” ( McKendree and Snowling, 2011).

McKendree & Snowling’s statement, “..if students with dyslexia are at a disadvantage, this should show up in the early years of the curriculum”( McKendree and Snowling, 2011) could, possibly, reflect an outdated view of the struggles associated with dyslexia and the settings in which these students under-perform. Their study excludes the later, clinically oriented years, thus, possibly excluding important aspects of SWD’s educational experiences.

The above studies all draw conclusions from performance within summative examinations. Little reference is made to performance within clinical settings or across courses in their entirety.

## Discussion

We identified three papers, which studied medical students with dyslexia in the UK. These all examined the impacts of allowing extra time (“compensations”) for SWD in examinations. It was not possible to conduct a meta-analysis of their findings because of the low number of studies and the high level of heterogeneity between them. Thus we summarised and reported their findings.

**MCQ:** Ricketts et al and McKendree & Snowling agreed that 25% extra time in MCQs allows students with dyslexia/SpLDs to perform as well as other students.

**EMQ** : Gibson & Leinster and McKendree & Snowling agreed that 25% extra time helped compensate for dyslexia. However, Gibson & Leinster noted that this did not help First-Year students in EMQs.

**SAQ:** Gibson & Leinster reported that SWD were particularly disadvantaged in SAQs without the extra 25% time.

**MEQ:** McKendree & Snowling reported that 25% extra time allowed SWD to perform equally well in MEQs.

**OSCE:** Gibson & Leinster reported that 25% extra time allowed SWD to perform equally well in OSCEs. However, McKendree & Snowling found no differences between SWD and students without dyslexia in their scores on performance-based OSCEs.

Within the wider literature concerning dyslexia in medical students there are also some notable gaps. There were no published studies on the emotional impacts of studying medicine with dyslexia. As a potentially stigmatising condition, this is an important area. Studies of nursing students have explored this area and found disturbing results - for example, that students felt stigmatised and unwanted on clinical placements ( Morris and Turnbull, 2006, Child and Langford, 2011). Whilst there are no similar published studies of medical students, this does correspond with our own findings on the experiences of UK junior doctors ( Shaw & Anderson, 2017). Another area lacking in research is the disclosure habits and beliefs of medical SWD. It is known that medical students tend not to disclose “disabilities” in general ( Miller et al., 2009). Many medical students are, in fact, not aware of their learning difficulties until they arrive at medical school ( Rosebraugh, 2000). Whilst dyslexia in itself may not be considered a disability, it is possible that SWD may still be emotionally adjusting to their new label when they first enter the clinical setting. The workloads and assessment burdens within medical schools emphasise the importance of further investigation to explore the experiences of medical SWD and how educational supports may impact upon their performance.

There were also no studies investigating how SWD perform within medical school entrance examinations, or how Widening Access programmes may effect their participation within medicine. Nor could we find any studies relating to performance across medical school in its entirety.

Finally, our discussion could not be complete without the reference to the support of medical SWD. There is currently no research into what supportive measures medical SWD would value, nor what proves to be most effective. The studies we found concluded that extra time in exams may be effective at removing any disadvantage caused by dyslexia. But there is still a need for further studies of different exam formats, and different supportive measures.

### Limitations

We did not locate or review any unpublished data. It is possible that some unknown studies are missing from this review. The shortage of research in this area also limits the topics discussed. This is reflected by our inability to conduct a meta-analysis and the high level of heterogeneity between studies. Finally, the first author of this review has dyslexia. This may have unknowingly introduced bias into our interpretation of the existing research.

## Conclusions

Medical SWD may underperform early within the degree, but their performance has been shown to equalise with other students as the course progresses - particularly as compensations are applied. Exams have been shown to not disadvantage medical SWD, so long as extra time is allowed. These statements are, however, based on 3 single-centre studies.

There is a paucity of research in this area. There are only three recent high quality quantitative studies. There are two other (American) studies - one idiosyncratic, and a single-case study - exploring the diagnostic rates and procedures for medical students and doctors referred for dyslexia assessments ( Banks et al., 1995, Guyer, 1988). There is a need for studies of admissions procedures, exams and supports for SWD within medical education. The studies reviewed here are a start, but one must bear in mind the relatively small numbers of SWD included in them.

We are in the process of conducting research in this field. Our first step was an autoethnographic study, published elsewhere ( Shaw et al., 2016). This was followed by a phenomenological study into the lived experiences of medical students and junior doctors with dyslexia - the results concerning the experiences of junior doctors have recently been published ( Shaw & Anderson, 2017). This led to the development of an online survey questionnaire to test the generalizability of those findings (on-going).

## Take Home Messages

• There has been little research into medical students with dyslexia.
• Medical students with dyslexia may perform on par with their non-dyslexic peers in exams, when supportive measures are in place.
• Medical students with dyslexia may, however, be slow to adapt to the medical school environment and struggle early on in their studies.
• The evidence on which this review is based is, however, limited. A useful meta-analysis was also not possible.
• There is a need for further research into the impact of dyslexia on medical studies in general.

## Notes On Contributors

**Sebastian Shaw** has an MSc in Medical Education. His main interests are Specific Learning Difficulties, and the psychosocial aspects of the student/trainee experience. He has several years of experience in researching these areas. He is a Member of the Academy of Medical Educators, and an Associate Fellow of the Higher Education Academy.

**Muzaffar Malik** is medical doctor by training, with an MSc in Public Health. He worked for over 10 years in this field in both national and international organizations, including the World Health Organization and the UK National Health Service. His ongoing academic career began in 2006.

**John Anderson** is a medical sociologist. His career has mainly been in teaching and research in medical schools. He is a Senior Fellow of the Higher Education Academy and is currently a Principal Lecturer in Postgraduate Medicine, within the Department of Medical Education at Brighton and Sussex Medical School.

## Declarations

The first author of this review has dyslexia.
