# Supplementary material for: The exam performance of medical students with dyslexia: a review of the literature
Source: MedEdPublish (2016). 2017 Jul 3;6:116. [Version 1] doi: 10.15694/mep.2017.000116 (PMC10885271; doi:10.15694/mep.2017.000116)
Supplement: Peer review history

# Reviewer report: Reviewer response for version 1

**DOI:** 10.21956/mep.19303.r28141

**Published:** 2017-07-24

**Authors:** Trudie Roberts

**Affiliations:** 1 Leeds Institute of Medical Education

This review has been migrated. The reviewer awarded 4 stars out of 5 This is a very interesting and useful paper about an important issue that all medical schools are challenged with. It would have been even better if the authors could have reviewed the literature outside medicine as although medical studies do pose particular issues and have specialised assessments it would have been good to know what we can learn from the wider literature - perhaps their next paper.As the authors note we do need more research in this area

Reviewer Expertise:

NA

No decision status is available

# Reviewer report: Reviewer response for version 1

**DOI:** 10.21956/mep.19303.r28142

**Published:** 2017-07-17

**Authors:** Gert Van Zyl

**Affiliations:** 1 University of the Free State

This review has been migrated. The reviewer awarded 4 stars out of 5 A very important paper on a very important topic. Well researched although as mentioned the numbers may be on the low side. Very important as a consideration in assessment practices and how to deal with this challenge and to accommodate these type of students. Further research and reporting encouraged in a very important area!

Reviewer Expertise:

NA

No decision status is available

# Reviewer report: Reviewer response for version 1

**DOI:** 10.21956/mep.19303.r28139

**Published:** 2017-07-13

**Authors:** Trevor Gibbs

**Affiliations:** 1 AMEE

This review has been migrated. The reviewer awarded 4 stars out of 5 An interesting and important paper from a group of authors who have published before on this subject. Sadly the lack of papers in the specific area of the authors' search reflect perhaps the importance of this subject in medical schools and curriculum designers eyes, maybe related to the apparent low number of medical / healthcare students with dyslexia. I would however fully support the authors' views that this is probably too low a number and that more work in this area will not only increase this number but also make clearer how we deal with the issue.I wish the authors well in there important research in the area

Reviewer Expertise:

NA

No decision status is available

# Reviewer report: Reviewer response for version 1

**DOI:** 10.21956/mep.19303.r28140

**Published:** 2017-07-07

**Authors:** James Fraser

**Affiliations:** 1 Griffith University

This review has been migrated. The reviewer awarded 5 stars out of 5 Through consideration of the impact of dyslexia on examination performance and how to structure reasonable adjustments to traditional assessment formats the authors are addressing the important topic of supporting students who have learning disabilities during their medical studies. To date this has received limited attention in published literature. The contrast between the population incidence of dyslexia and that of medical students is interesting and raises questions relating to the impact of selection process, about perceptions of stigmatisation and non-disclosure of learning disabilities as a result and challenges medical programs to ensure appropriate student support processes exist and are utilised by students. This paper also challenges us to think about our assessment format and design and how we ensure that they equitable for all students, and whether some students can be assessed using alternate assessment formats to address the desired learning outcomes. It would be great to see further collaborative work in this area.

Reviewer Expertise:

NA

No decision status is available
